# SP1-Mediated Upregulation of Long Noncoding RNA ZFAS1 Involved in Non-syndromic Cleft Lip and Palate *via* Inactivating WNT/β-Catenin Signaling Pathway

**DOI:** 10.3389/fcell.2021.662780

**Published:** 2021-06-29

**Authors:** Shiyu Chen, Zhonglin Jia, Ming Cai, Mujie Ye, Dandan Wu, Teng Wan, Bowen Zhang, Peixuan Wu, Yuexin Xu, Yuntao Guo, Chan Tian, Duan Ma, Jing Ma

**Affiliations:** ^1^Institutes of Biomedical Sciences, Fudan University, Shanghai, China; ^2^ENT Institute, Department of Facial Plastic and Reconstructive Surgery, Eye & ENT Hospital, Fudan University, Shanghai, China; ^3^Center for Reproductive Medicine, Department of Obstetrics and Gynecology, National Clinical Research Center for Obstetrics and Gynecology, Ministry of Education, Beijing, China; ^4^Beijing Key Laboratory of Reproductive Endocrinology and Assisted Reproductive Technology, Peking University Third Hospital, Beijing, China; ^5^Key Laboratory of Assisted Reproduction, Peking University, Beijing, China; ^6^State Key Laboratory of Oral Diseases and National Clinical Research Center for Oral Diseases and Department of Cleft Lip and Palate, West China Hospital of Stomatology, Sichuan University, Chengdu, China; ^7^Department of Oral and Cranio-Maxillofacial Surgery, Shanghai Ninth People’s Hospital, College of Stomatology, Shanghai Jiao Tong University School of Medicine, Shanghai, China; ^8^School of Basic Medical Sciences, Fudan University, Shanghai, China; ^9^Children’s Hospital of Fudan University, Shanghai, China; ^10^Medical Laboratory of Nantong ZhongKe, Nantong, China

**Keywords:** NSCLP, ZFAS1, Sp1, CTCF, Wnt/β-catenin pathway

## Abstract

Non-syndromic cleft lip and palate (NSCLP) is one of the most common congenital malformations with multifactorial etiology. Although long non-coding RNAs (lncRNAs) have been implicated in the development of lip and palate, their roles in NSCLP are not fully elucidated. This study aimed to investigate how dysregulated lncRNAs contribute to NSCLP. Using lncRNA sequencing, bioinformatics analysis, and clinical tissue sample detection, we identified that lncRNA ZFAS1 was significantly upregulated in NSCLP. The upregulation of ZFAS1 mediated by SP1 transcription factor (SP1) inhibited expression levels of Wnt family member 4 (*WNT4*) through the binding with CCCTC-binding factor (CTCF), subsequently inactivating the WNT/β-catenin signaling pathway, which has been reported to play a significant role on the development of lip and palate. Moreover, *in vitro*, the overexpression of ZFAS1 inhibited cell proliferation and migration in human oral keratinocytes and human umbilical cord mesenchymal stem cells (HUC-MSCs) and also repressed chondrogenic differentiation of HUC-MSCs. *In vivo*, ZFAS1 suppressed cell proliferation and numbers of chondrocyte in the zebrafish ethmoid plate. In summary, these results indicated that ZFAS1 may be involved in NSCLP by affecting cell proliferation, migration, and chondrogenic differentiation through inactivating the WNT/β-catenin signaling pathway.

## Introduction

Cleft lip and palate (CLP) is one of the most common congenital craniofacial malformations around the world, resulting from failure in fusion of the facial primordia. Even though surgical treatments are doing well, children with CLP still face a variety of postoperative complications and psychosocial problems (Dixon et al., 2011).

There are two forms of CLP, syndromic and non-syndromic CLP (NSCLP). Syndromic CLP was often caused by single-gene mutations and chromosomal abnormalities. NSCLP, accounting for 70% of CLP, is a disorder without additional symptoms of atypical conditions (Jiang et al., 2006; Leslie and Marazita, 2013). It arises in about one per 500–1,000 live births (Mohamad Shah et al., 2019). NSCLP is a complex disease with multiple factors including genetic, epigenetic, and environmental factors. A number of susceptibility genes for NSCLP have been identified in previous studies (Dixon et al., 2011). However, only a small minority of NSCLP results from single-gene mutations. Because proper development of the lip and palate requires a coordination of a series of complex events, including cell growth, migration, differentiation, and apoptosis (Leslie and Marazita, 2013), which are regulated by precise gene expression, NSCLP could also be associated with the disturbances in this process due to dysregulation of genes.

Epigenetic modifications play an important role in the regulation of gene expression. As an important component of epigenetic modifications, long non-coding RNAs (lncRNAs), consisting of over 200 nucleotides, are non-coding transcripts and numerous in eukaryotes (Ma et al., 2020a; Ye et al., 2020b). They were thought to be “noise” in genome and a byproduct of transcription. Nevertheless, recent studies have revealed that lncRNAs are involved in several biological processes (BPs) through regulating gene expression at epigenetic, genomic transcription, and post-transcriptional levels (Ye et al., 2019; Ma et al., 2020b). Moreover, dysregulation of lncRNAs could be related to the progression of many disorders (Peng et al., 2017; Chi et al., 2019; Ye et al., 2020a). They have also been shown to participate in the development of lip and palate and the pathogenesis of related diseases (Gerrard et al., 2016; Gao et al., 2019; Shu et al., 2019). There have been a few of reports on the role of lncRNAs in CLP (Uslu et al., 2014; Gao et al., 2017; Yun et al., 2019), let alone NSCLP. RNA sequencing to identify transcriptome profiles including mRNAs, lncRNAs, and microRNAs (miRNAs) in NSCLP was performed in 2019 for the first time, and the potential role of lncRNAs in NSCLP has been suggested (Gao et al., 2019). So far, only several lncRNAs have been identified to be associated with NSCLP in previous studies, including lncRNA RP11-462G12.2, CCDC26 lncRNA, and a long non-coding interval at 8q24 (Uslu et al., 2014; Wu et al., 2019; Yun et al., 2019). Therefore, the underlying mechanism and function of lncRNAs in the involvement of NSCLP remain largely unclear.

In the present study, we identified the lncRNA profiles of tissue samples from patients with NSCLP using lncRNA sequencing followed by a series of bioinformatics analysis, and then we focused on ZFAS1 with unknown function in CLP. ZFAS1 was significantly upregulated in patients with NSCLP. *In vitro*, the overexpression of ZFAS1 significantly suppressed cell proliferation, cell migration, and chondrogenic differentiation. *In vivo*, upregulation of ZFAS1 in zebrafish embryos decreased cell proliferation and numbers of chondrocyte in the ethmoid plate. Exploration for further molecular mechanism suggested that ZFAS1 mediated by transcription factor SP1 inactivated the WNT/β-catenin signaling pathway through the binding with CCCTC-binding factor (CTCF).

## Materials and Methods

### Study Subjects and Samples

This study was reviewed and approved by the Institutional Research Ethics Committee of the Children’s Hospital of Fudan University (2016-121). A total of 40 tissues from patients with NSCLP and 15 normal control (NC) tissues, which were the extra tissues from surgery, were obtained from the Children’s Hospital of Fudan University. None of the patients with NSCLP had been diagnosed with extra disorders. NC samples were from patients with a traumatism for surgery and without any congenital disorders. All samples for RNA extraction were maintained in RNAlater RNA Stabilization Solution (Thermo Fisher Scientific, Waltham, MA, United States) after surgery and were stored at −80°C.

### Long Non-coding RNA Sequencing and Functional Enrichment Analysis

Long non-coding RNAs sequencing of tissue samples from four NSCLP and two NC was performed by Precisiongenes (Nantong, China) to identify the differentially expressed lncRNAs. In brief, ribosomal RNA (rRNA) in total RNA was removed, and the remaining RNAs were subjected to construct sequencing library. The library was then sequenced on an Illumina platform (Illumina, Inc., San Diego, CA, United States), and the trimmed reads were mapped to the human genome Hg38. Finally, the transcript read count was calculated. The DAVIDdatabase^1^ was applied to perform gene ontology (GO) and Kyoto Encyclopedia of Genes and Genomes (KEGG) pathway analyses on the differentially expressed lncRNAs. *P* < 0.05 was considered to be statistically significant.

### RNA Extraction, Reverse Transcription, and Quantitative Polymerase Chain Reaction

Total RNA was isolated from tissues or cells using TRIzol reagent (Invitrogen, Carlsbad, CA, United States) according to the manufacturer’s protocol. Reverse transcription of 1,000 ng of RNA was performed to synthesize cDNA using a Primer Script RT Reagent Kit (Takara, Tokyo, Japan), followed by quantitative polymerase chain reaction (qPCR) using SYBR Premix Ex^TM^ Taq (Takara) on a StepOnePlus^TM^ Real-Time PCR system (Thermo Fisher Scientific). Gene expression was normalized to glyceraldehyde 3-phosphate dehydrogenase (GAPDH) and calculated by the relative expression method (2^–ΔΔCt^). The primers used were as follows:

ZFAS1-Q-F: TCTCCTAGTTGCAGTCAGGC,

ZFAS1-Q-R: ATGCGGGTGTTGGAAGTAGA;

GAPDH-Q-F: GGGAGCCAAAAGGGTCAT,

GAPDH-Q-R: GAGTCCTTCCACGATACCAA;

U1-Q-F: GACGGGAAAAGATTGAGCGG,

U1-Q-R: CCACGAAGAGAGTCTTGAAGG;

Actin-Q-F: CATGTACGTTGCTATCCAGGC,

Actin-Q-R: CTCCTTAATGTCACGCACGAT;

Sox9-Q-F: ATGAAGATGACCGACGAGCA,

Sox9-Q-R: AACTTGTCCTCCTCGCTCTC;

ACAN-Q-F: CTTCTGAGTTCGTGGAGGGT,

ACAN-Q-R: CGGACGTCTCACTGCTAGAT;

SP1-Q-F: TCCAGACCATTAACCTCAGTGC,

SP1-Q-R: ACCACCAGATCCATGAAGACC;

WNT4-Q-F: GCTGTGACAGGACAGTGCAT,

WNT4-Q-R: GCCTCATTGTTGTGGAGGTT.

### Cell Culture

Human oral keratinocyte (HOK) cells were cultured in Dulbecco’s modified Eagle’s medium (Biological Industries, Beit HaEmek, Israel) with 10% fetal bovine serum (FBS) (Biological Industries) and 1% penicillin–streptomycin (Biological Industries) at 37°C in 5% CO_2_.

Human umbilical cord mesenchymal stem cells (HUC-MSCs) were cultured in Dulbecco’s modified Eagle’s medium/F12 (Gibco, Carlsbad, CA, United States) with 10% FBS (Gibco) and 1% penicillin–streptomycin (Biological Industries) at 37°C in 5% CO_2_. The expanded cells at passages 3–7 were used for experiments.

All cell culture dishes and culture plates were purchased from Hangzhou Xinyou Biotechnology Co., Ltd. (Hangzhou, China).

### Plasmid Construction

The plasmids of ZFAS1-PCDH and SP1-PCDH were obtained from GeneRay (Shanghai, China). The plasmids were sequenced and shown to be consistent with the sequence in the National Center for Biotechnology Information database^2^. The recombinant luciferase plasmids (pGL3-basic-ZFAS1-E1-WT; pGL3-basic-ZFAS1-E2-WT; and pGL3-basic-ZFAS1-E1-Del1/pGL3-basic-ZFAS1-E1-Del2) were constructed with the corresponding sequences amplified from human genomic DNA. Short hairpin RNAs (shRNAs) targeting ZFAS1 and SP1 were designed and cloned into the lentiviral vector pGreen. Empty vector was used as a negative control.

The shRNA sequences used were as follows:

ZFAS1-shRNA: GATCCGCTATTGTCCTGCCCGTTAGAG TTCAAGAGACTCTAACGGGCAGGACAA TAGCTTTTTTG;

SP1-shRNA: CCGGGCTGGTGGTGATGGAATACATCT CGAGATGTATTCCATCACCACCAGCTTTTT.

### Cell Proliferation Assay

Cell proliferation assay was detected using Cell Counting Kit-8 (CCK8; Dojindo, Tokyo, Japan) according to the manufacturer’s instructions. Cells were seeded into 96-well plates at 1 × 10^4^ cells/well. After cell culture for 0, 24, 48, and 72 h, 10 μl of CCK8 solution was added to each well and incubated at 37°C for 3 h. The absorbance was measured at a wavelength of 450 nm for each well. All samples were prepared in triplicate and normalized to blank controls.

### Cell Cycle Analysis

Cells were harvested and fixed with 75% ethanol at 4°C overnight. And cells were washed with phosphate-buffered saline (PBS) for three times, followed by incubation with RNA enzyme containing propidium iodide (PI; 40%) (Sigma-Aldrich, St. Louis, MO, United States) for 30 min at room temperature. Cell cycle was detected by FACS Calibur (BD Biosciences, San Jose, CA, United States), and data analysis was performed by FACS Diva (BD Biosciences).

### Transwell Assay

Cells were starved in serum-free medium overnight, and then a cell suspension of 200 μl of serum-free medium was seeded into each well of the upper transwell chamber (Corning, New York, NY, United States). In the lower chamber, 750 μl of medium with 25% FBS was added. Through incubation at 37°C for 48 h, cells on the upper surface were removed by a cotton tip. Cells on the lower surface were fixed with 4% paraformaldehyde (PFA) and stained with 0.1% crystal violet for 30 min. The results were observed by EVOS^TM^ Microscope M5000 Imaging System (Invitrogen).

### Nuclear–Cytoplasmic Separation

Nuclear and cytoplasmic fractions were isolated from cells using PARIS kit (Thermo Fisher Scientific) according to the manufacturer’s protocols. Actin was used as a cytoplasmic control, and U1 was used as a nuclear control. RNA levels of ZFAS1, actin, and U1 in the cytoplasm and nuclear fractions were quantified by qPCR.

### RNA Fluorescence *in situ* Hybridization Assay

Cells were seeded in a 24-well plate for 24 h and fixed with 4% PFA for 20 min. The cells in the plate were pre-hybridized with a hybridization buffer at 42°C for 1 h and overnight with hybridization reaction solution containing the fluorescence *in situ* hybridization (FISH) probe in the dark. The nonspecific probe was removed with 2× saline sodium citrate (SSC) containing 50% formamide for 10 min at 37°C, 1× SSC for 2 × 5 min at 37°C, and 0.5× SSC for 10 min at room temperature. The anti-biotin monoclonal antibody and secondary antibody were used for detecting biotin-labeled ZFAS1. Cells in the plates were incubated with DAPI (Cell Signalling Technology, Boston, MA, United States). Finally, the cells were subjected to fluorescence signal detection under an EVOS^TM^ Microscope M5000 Imaging System (Invitrogen). The probe of ZFAS1 was synthesized in Guangzhou RiboBio Co., Ltd. (Guangzhou, China).

### *In vitro* Chondrogenic Differentiation

High-density pellet cultures were performed with HUC-MSCs at passages 3–7. To form a cell pellet, 2 × 10^5^ HUC-MSCs were centrifuged in a 15-ml polypropylene conical tube at 1,000 rpm for 5 min. And pellets were incubated in the StemPro chondrogenesis differentiation medium (Gibco) at 37°C in 5% CO_2_ for 7/14 days. The differentiation medium was changed every 3 days.

### Histology Staining and Immunohistochemistry

The cell pellets were fixed in 4% PFA for 24 h, dehydrated in graded series of ethanol (70–100%), processed with xylene (50–100%), and then embedded in paraffin. Paraffin blocks were cut into consecutive sections using a microtome and placed onto glass slides. Following deparaffinization, the sections were used for subsequent experiments.

For Alcian blue staining, the pellets sections were treated with 1% Alcian blue (Sigma-Aldrich) dissolved in acetic acid for 1 h. For toluidine blue staining, the sections were incubated with toluidine blue solution (Sigma-Aldrich) for 1 h. These staining sections were observed in EVOS^TM^ Microscope M5000 Imaging System (Invitrogen).

The pellet sections were subjected to immunohistochemistry (IHC). The antibody used was Collagen type II (1:100; Affinity Biosciences, Cincinnati, OH, United States). Antigen was retrieved by pepsin (Sigma-Aldrich) for 10 min, and 5% goat serum was applied to block the unspecific background antigen. Sections were incubated with the primary antibodies overnight at 4°C. According to the manufacturer’s instructions, a streptavidin–horseradish peroxidase (HRP) detection system (MxBiotech, Eugene, OR, United States) was applied, followed by hematoxylin staining and neutral balsam sealing. These sections were observed in EVOS^TM^ Microscope M5000 Imaging System (Invitrogen).

### Zebrafish Breeding and Husbandry

The Institutional Research Ethics Committee of the Children’s Hospital of Fudan University, China, approved and monitored all zebrafish procedures following the guidelines and recommendations outlined by the Guide for the Care and Use of Laboratory Animals (2016-121). Zebrafish were raised at 28°C under a condition of 14-h light/10-h dark with twice-a-day feeding at the Fudan University. The *Tg(col2a1a-mcherry)* zebrafish, which is cartilage specific and labeled with red fluorescence was purchased from Nanjing YSY Biotech Company Ltd (Nanjing, China). Adult zebrafish were acclimated to laboratory conditions for 4 weeks prior to breeding for further experiments.

### Microinjection

Full-length lncRNAs were *in vitro* transcribed using a mMESSAGE mMACHINE Kit (Ambion, Austin, TX, United States) with the dsDNA templates coupled with SP6 promoter at 37°C overnight. After *in vitro* transcription, the DNA templates were removed by RNase-free DNase at 37°C for 15 min, followed by purification with RNAclean Kit (Tiangen, Beijing, China). At the night before mating, the males and females were separated by a transparent wall. The following morning, the transparent wall was removed, and the water was changed with warm water at temperature of 29°C. LncRNAs were diluted to 300 ng/μl using RNase-free water for microinjection of experimental group. RNase-free water was for the control group. Embryos were collected 20 min after spawning. Then microinjection into single-cell regions of embryos was performed with a Narishige IM-300 microinjector (Narishige Laboratory Instruments Ltd., Tokyo, Japan). Later, embryos were incubated in the E3 medium at 28°C. Unfertilized eggs and dead embryos were removed immediately, and E3 medium was refreshed every day. And then these embryos were used for further experiments and observation.

### Bromodeoxyuridine Assay

Zebrafish embryos were incubated in E3 medium containing 10 mM of bromodeoxyuridine (BrdU) for 6 h, and then embryos were fixed with 4% PFA for 4 h, followed by 0.2 N of HCl for 1 h at room temperature. Anti-BrdU antibody (1:100; Santa Cruz Biotechnology, Paso Robles, CA, United States) and DAPI (1:100) were incubated overnight at 4°C. Embryos were washed with PBS and incubated with Goat Anti-Mouse IgG488 (1:200; Yisheng, Shanghai, China) overnight at 4°C. Histochemical detection was performed by Olympus MVX10 microscope (Olympus, Tokyo, Japan).

### RNA Pull-Down Assay and Mass Spectrometry Analysis

Biotinylated ZFAS1 sense and antisense RNA were transcribed using Large Scale RNA Production System-T7 (Promega, Madison, WI, United States) and RNA 3′ End Desthiobiotinylation Kit (Thermo Fisher Scientific) *in vitro*. About 1 × 10^7^ cells were dissolved in cell lysis buffer with RNasin (Promega). RNA pull-down assay was conducted through Pierce^TM^ Magnetic RNA-Protein Pull-Down Kit (Thermo Fisher Scientific). In brief, the biotinylated ZFAS1 sense and antisense RNA were incubated with Pierce Nucleic-Acid Compatible Streptavidin Magnetic Beads and total cell lysates at room temperature for 2 h. Finally, the retrieved proteins were detected by sodium dodecyl sulfate–polyacrylamide gel electrophoresis (SDS-PAGE) gels for mass spectrometry analysis. Primers used are as follow:

ZFAS1-sense-F: TAATACGACTCACTATAGGGCCACGC TGTTTTGACCTCCA,

ZFAS1-sense-R: GAGCGATCGCAGATCCTTCG;

ZFAS1-antisense-F: CCACGCTGTTTTGACCTCCA,

ZFAS1-antisense-R: TAATACGACTCACTATAGGGGAGC GATCGCAGATCCTTCG.

### RNA Immunoprecipitation Assay

About 1 × 10^7^ cells were harvested and lysed with RNA immunoprecipitation (RIP) lysis buffer at −80°C overnight. Five micrograms of human anti-CTCF (Abcam, Cambridge, United Kingdom) or NC rabbit anti-IgG antibody (ProteinTech, Chicago, IL, United States) were incubated with magnetic beads at room temperature for 1 h and then incubated with cell lysates at 4°C overnight. RNase-free DNase I (Promega) and proteinase K (Yeasen, Shanghai, China) were used for digesting extra DNA and proteins, respectively. Finally, the retrieved RNA was subjected to qRT-PCR analysis of ZFAS1. Total RNA (input controls) and rabbit IgG controls were measured simultaneously to normalize the binding capacity between RNA and protein.

### Western Blotting and Antibodies

Cells were harvested and lysed with radioimmunoprecipitation assay (RIPA) buffer (Yeasen) containing a protease inhibitor cocktail (Sigma-Aldrich). Proteins were quantified by a Bradford Protein Assay Kit (Abcam), and the same amounts of proteins were separated by 10% SDS-PAGE and transferred to nitrocellulose membranes (Pall, Port Washington, NY, United States), followed by a block with 8% milk dissolved in Tris-buffered saline Tween for 1 h at room temperature. And then the membranes were incubated with specific primary antibodies at 4°C overnight, followed by secondary antibodies at room temperature for 1 h. Antibodies used were as follows: CTCF (1:5,000, Abcam), GAPDH (1:5,000, ProteinTech), WNT4 (1:1,000, Santa Cruz), β-catenin (1:1,000, Cell Signalling Technology), and c-Myc (1:5,000, ProteinTech).

### Data Mining of Chromatin Immunoprecipitation Sequencing Data

Chromatin immunoprecipitation sequencing (ChIP seq) data of CTCF were mined and selected in Cistrome Data Browser^3^ based on the following requirements: (1) species, *Homo sapiens*; (2) cell type, HEK293T cells; (3) factors, CTCF; and (4) quality control meets the basic requirements. GSM2026782 was selected for further analysis.

### RNA Sequencing

A group of HOK cells overexpressing ZFAS1 and a group of NC cells were subjected to RNA sequencing by GENEWIZ (Suzhou, China). In brief, RNA sequencing processes included RNA extraction, RNA sample quality detection, library construction/purification/detection/quantification, generation of sequencing cluster, and sequencing. And then the results were subjected to perform GO/KEGG analysis.

### Chromatin Immunoprecipitation Assay

Cells were crosslinked with 1% formaldehyde and quenched in glycine solution. ChIP assay was performed by Pierce Magnetic Chip Kit (Thermo Fisher Scientific), according to the manufacturer’s instructions. Two micrograms of anti-CTCF antibody (Abcam) and normal rabbit IgG (ProteinTech) were used for immunoprecipitation. ChIP-enriched DNA samples were quantified by qPCR to determine the CTCF binding sites of WNT4 genome. The value was shown as relative enrichment normalized to IgG. Primers used for ChIP-qPCR were as follows:

WNT4-peak-Q-F: TGACAGGTCGCTGAGGAAG,

WNT4-peak-Q-R: CCCCAAGCAGAAACAGGTC.

### Dual-Luciferase Reporter Assay

The recombinant plasmid pGL3-basic-ZFAS1-E1-WT/del1/del2 was constructed. Cells were seeded in 96-well plates at 1 × 10^4^ cells per well and incubated at 37°C overnight. SP1 was co-transfected into cells with pGL3-basic-ZFAS1-E1-WT/del1/del2 using Lipofectamine 3000 (Invitrogen). Cells were collected 48 h after transfection. Both firefly and Renilla luciferase activities were measured by a Dual-Luciferase Reporter Assay (Promega). And the firefly luciferase activities were normalized to Renilla luciferase activities.

### Statistical Analysis

All experiments were repeated three times. All statistical analyses were conducted with paired two-tailed Student’s *t*-test with GraphPad Software Inc. (La Jolla, CA, United States). *P* < 0.05 was considered significant.

## Results

### ZFAS1 Was Screened Through Long Non-coding RNA Sequencing and Further Bioinformatics Analysis

**FIGURE 1:**
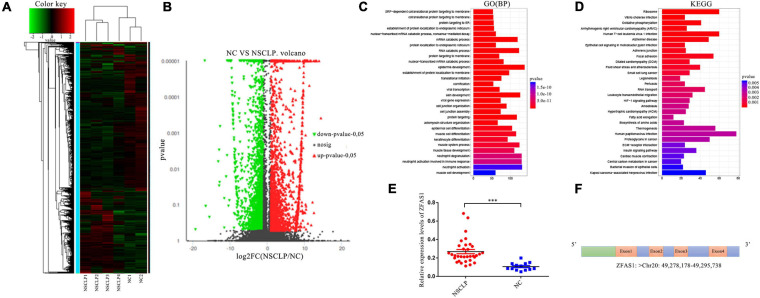
ZFAS1 was screened through long non-coding RNA (lncRNA) sequencing and further qPCR assay. **(A)** Heatmap of dysregulated lncRNAs identified from lncRNA sequencing by hierarchical clustering analysis between the non-syndromic cleft lip and palate (NSCLP) (NSCLP1/2/3/4) and normal control (NC) (NC1/2) samples. Expression values are represented by red and green shades, indicating expressions above and below the median expression level across all samples, respectively. **(B)** The volcano plot of differentially expressed lncRNAs. The fold change threshold is 1.5, and *P*-value ≤ 0.05. The red dots indicate upregulated genes, the green dots indicate downregulated genes, and the black dots indicate non-differentially expressed genes. **(C,D)** The gene ontology (GO) and Kyoto Encyclopedia of Genes and Genomes (KEGG) pathways significantly enriched in the differentially expressed lncRNAs. The length and color of the bar represent the amount of differentially expressed lncRNAs enriched in the pathway and enrichment significance, respectively. BP, biological processes. **(E)** ZFAS1 was upregulated in tissues from NSCLP compared with NC tissues by qPCR assay. **(F)** The schema of ZFAS1 in chromosome. Values are mean ± SEM, *n* = 3, ***P < 0.0001.

A total of 349 lncRNAs were significantly dysregulated, with 59 upregulated lncRNAs and 290 downregulated lncRNAs in NSCLP by lncRNA sequencing and bioinformatics analysis (Figures 1A,B). The GO pathway analysis revealed these differentially expressed lncRNAs were distinctly enriched in BP terms such as “epidermis development,” “skin development,” and “protein targeting” (Figure 1C). And the KEGG pathway analysis showed these lncRNAs are enriched in “Ribosome,” “Focal adhesion,” and so on (Figure 1D). Among the differentially expressed lncRNAs, we then selected 10 lncRNAs for expanded sample qPCR validation (Table 1), according to the following conditions: (1) log_2_ (FoldChange) > 1.5, *P* < 0.05; (2) the pathways that the lncRNA may be involved in are related to CLP based on enrichment analysis; and (3) the function of the lncRNA is associated with CLP by literature retrieval.

**TABLE 1:** Basic information of 10 lncRNAs selected from lncRNA sequencing.

Gene symbol	Log_2_FC (NSCLP/NC)	Changing trend	LncRNA type
SNHG3	1.28	Up	LincRNA
ZFAS1	1.79	Up	Antisense
SVIL-AS1	5.98	Up	Antisense
DSG1-AS1	1.51	Up	Antisense
HCG22	3.81	Up	LincRNA
NEAT1	−1.34	Down	LincRNA
RP11-373D23.2	−1.38	Down	Sense intronic
RP1-79C4.4	−1.27	Down	LincRNA
TPT1-AS1	−1.22	Down	Antisense
RP11-423G4.10	−1.43	Down	Sense intronic

*FC, fold change; lncRNAs, long non-coding RNAs; NSCLP, non-syndromic cleft lip and palate; lincRNA, long intergenic non-coding RNA.*

### ZFAS1 Was Upregulated in Tissue Samples From Patients With Non-syndromic Cleft Lip and Palate

We detected expression levels of these 10 lncRNAs in 36 tissue samples from patients with NSCLP and 13 NC tissue samples using qPCR assay. In view of the non-conservation of lncRNAs and feasibility of animal experiment, we chose the upregulated lncRNA as the research target in principle. Finally, we selected ZFAS1, which was the most significantly upregulated lncRNA in NSCLP compared with NC (Figure 1E and Supplementary Figure 1). ZFAS1 was an lncRNA located at chromosome 20q13, with four exons (Figure 1F).

### Overexpression of ZFAS1 Inhibited Cell Proliferation and Cell Migration in Human Oral Keratinocyte Cells and Human Umbilical Cord Mesenchymal Stem Cells

**FIGURE 2:**
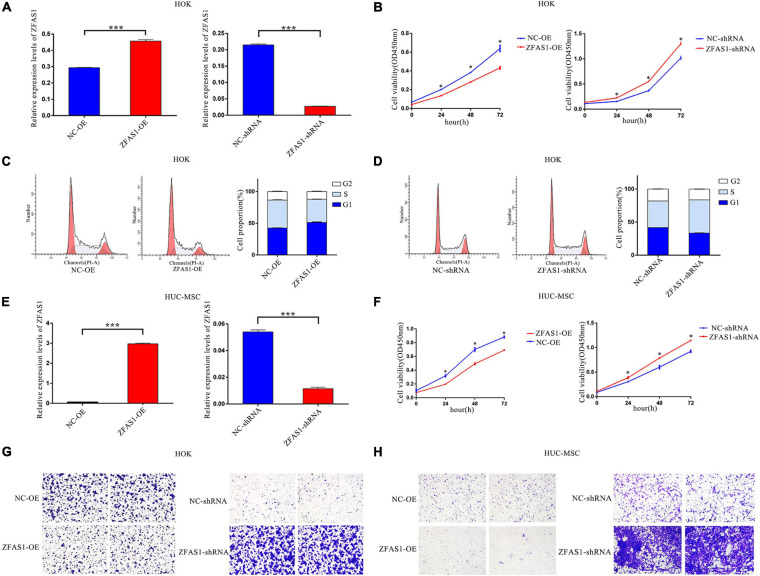
Overexpression of ZFAS1 inhibited cell proliferation and migration in human oral keratinocyte (HOK) cells and human umbilical cord mesenchymal stem cells (HUC-MSCs). **(A)** ZFAS1 was upregulated and downregulated in HOK cells using overexpression vector of ZFAS1 and shRNA targeting to ZFAS1, respectively. **(B)** Negative regulation of cell proliferation by ZFAS1 was revealed by Cell Counting Kit-8 (CCK8) assay in HOK cells. **(C,D)** ZFAS1 overexpression and knockdown in HOK cells increased and decreased G1/S arrest by cell cycle assay, respectively. The two left panels indicate the results from fluorescence-activated cell sorting (FACS), and the right panel indicates the statistical result of the two left panels. **(E)** ZFAS1 was upregulated and downregulated in HUC-MSCs using overexpression vector of ZFAS1 and shRNA targeting to ZFAS1, respectively. **(F)** Negative regulation of cell proliferation by ZFAS1 was revealed by CCK8 assay in HUC-MSCs. **(G,H)** Transwell assays revealed a negative regulation of cell migration by ZFAS1 in HOK cells and HUC-MSCs. Cells were stained with crystal violet. Values are mean ± SEM, *n* = 3, *P < 0.05, ***P < 0.0001. ShRNA, short hairpin RNAs; OE, overexpression.

To investigate the effects of ZFAS1 on cell phenotypes related to NSCLP, ZFAS1 was successfully upregulated and downregulated in HOK cells and HUC-MSCs (Figures 2A,E). HUC-MSCs have been identified by their special markers on a flow cytometry platform (Supplementary Figure 2). CCK8 assay showed that ZFAS1 overexpression significantly inhibited cell proliferation, consistent with the tendency in cells downregulating ZFAS1 (Figures 2B,F). Then cell cycle distribution of HOK cells presented that the G1 phase was much higher in cells overexpressing ZFAS1, indicating that the overexpression of ZFAS1 contributed to G1 phase arrest (Figures 2C,D). Transwell assay revealed that cell migration of HOK and HUC-MSCs was both significantly inhibited with the overexpression of ZFAS1, in accordance with the tendency in cells to downregulate ZFAS1 (Figures 2G,H). However, flow cytometry analysis showed that ZFAS1 dysregulation did not affect apoptosis in HOK cells (Supplementary Figure 3).

### ZFAS1 Overexpression Suppressed the Chondrogenic Differentiation Potential in Human Umbilical Cord Mesenchymal Stem Cells

**FIGURE 3:**
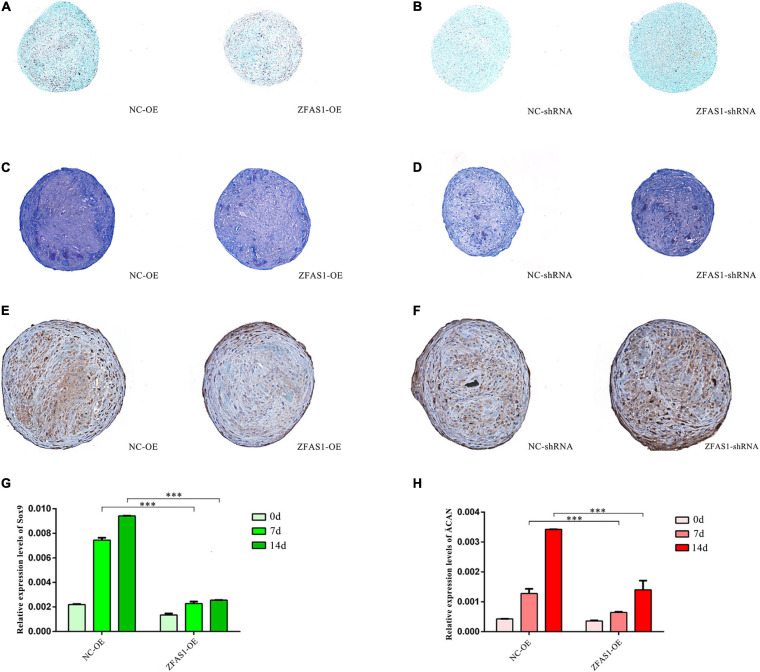
ZFAS1 overexpression suppressed the chondrogenic differentiation potential in human umbilical cord mesenchymal stem cells (HUC-MSCs). **(A,B)** Alcian blue staining of paraffin sections from chondrocyte pellets revealed a negative regulation of chondrogenic differentiation by ZFAS1. The color depth of Alcian blue indicates the levels of chondrogenic differentiation. **(C,D)** Toluidine blue staining of paraffin sections from chondrocyte pellets revealed a negative regulation of chondrogenic differentiation by ZFAS1. The color depth of toluidine blue indicates the levels of chondrogenic differentiation. **(E,F)** The immunohistochemistry (IHC) results of collagen type II in the paraffin sections from chondrocyte pellets. The brown regions represent the signal of collagen type II; the blue dots represent the signal of nuclei. **(G,H)** The qPCR assay showed that ZFAS1 overexpression downregulated the expression of Sox9 and ACAN in the chondrogenesis-induced pellets at 0/7/14 days. Values are mean ± SEM, *n* = 3, ***P < 0.0001. d, days.

Abnormal chondrogenic differentiation during palatogenesis may lead to NSCLP. To determine the biological effects of ZFAS1 on the chondrogenic ability, traditional high-density pellet cultures of HUC-MSCs were used for the induction of differentiation. At day 14 after cartilage pellet culture, both Alcian blue and toluidine blue staining showed that upregulating ZFAS1 significantly interfered with chondrogenesis (Figures 3A,C), while HUC-MSCs with ZFAS1 knockdown presented higher chondrogenesis potential (Figures 3B,D). By IHC in cartilage pellets, the expression of type II collagen, marker of chondrogenic matrix, was consistent with the results of staining (Figures 3E,F). The mRNA levels of chondrogenic markers SRY-box transcription factor 9 (Sox9) and aggrecan (ACAN) were significantly decreased in pellets overexpressing ZFAS1 at days 7 and 14, compared with NC (Figures 3G,H).

### ZFAS1 Overexpression in Zebrafish Embryos Significantly Decreased Cell Proliferation and Numbers of Chondrocyte in the Ethmoid Plate

**FIGURE 4:**
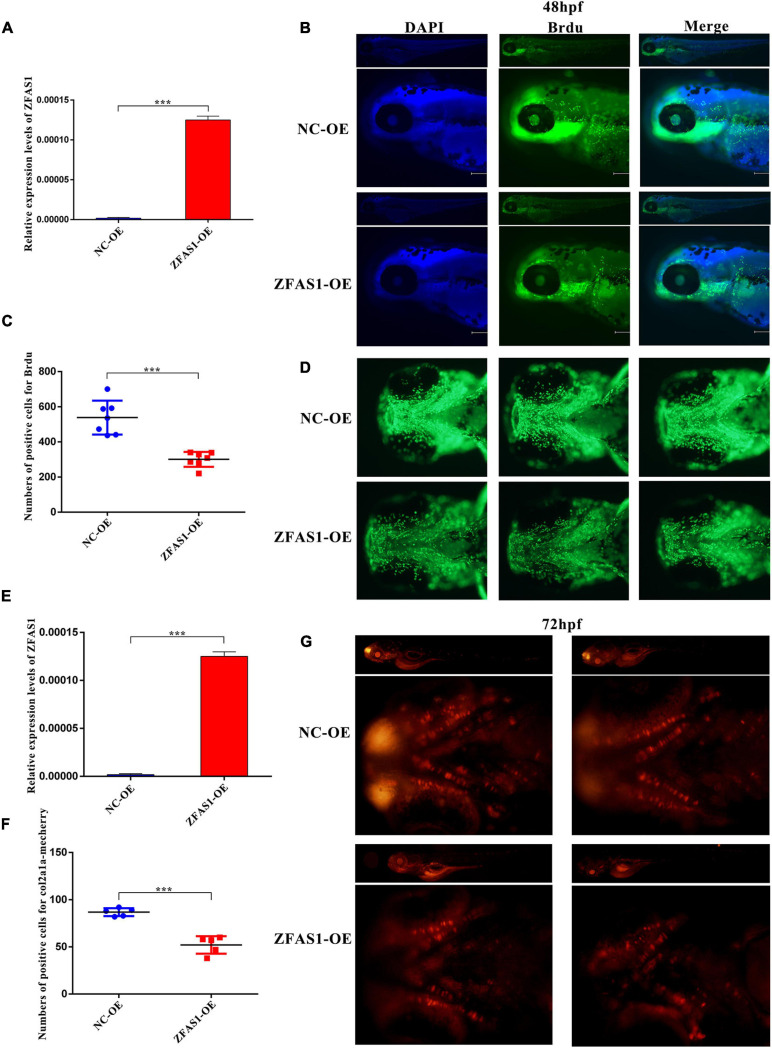
ZFAS1 overexpression in zebrafish embryos significantly decreased cell proliferation and numbers of chondrocyte in the ethmoid plate. **(A)** The efficiency of overexpressing ZFAS1 in wild-type zebrafish embryos by microinjection using the qPCR assay. **(B)** The side view of bromodeoxyuridine (BrdU)-labeling cells in zebrafish at 48 hpf detected by fluorescence microscope. Left, blue fluorescence for DAPI; middle, green fluorescence for BrdU; right, the merged image. **(C)** The statistical results of BrdU-positive cells in the zebrafish ethmoid plate. **(D)** The ventral view of BrdU-positive cells in the zebrafish ethmoid plate. **(E)** The efficiency of overexpressing ZFAS1 in *Tg(col2a1a-mcherry)* zebrafish embryos by microinjection using the qPCR assay. **(F)** The statistical results of col2a1a-mcherry-positive cells in the zebrafish ethmoid plate. **(G)** The ventral view of col2a1a-mcherry-positive cells in the zebrafish ethmoid plate at 72 hpf. Red fluorescence, col2a1a-mcherry. Values are mean ± SEM, *n* = 3, ***P < 0.0001.

To investigate the role of ZFAS1 in the palatogenesis, ZFAS1 was microinjected into zebrafish embryos to establish zebrafish overexpressing ZFAS1, and the efficiency was validated by qPCR at 48 h post-fertilization (hpf) (Figure 4A). The observation on the whole zebrafish indicated that microinjection did not affect the overall development of zebrafish, and BrdU assay demonstrated that the overexpression of ZFAS1 in zebrafish at 48 hpf can inhibit cell proliferation in the ethmoid plate (Figures 4B–D). To understand well the chondrocyte in the zebrafish ethmoid, ZFAS1 was overexpressed in the *Tg(col2a1a-mcherry)* zebrafish (Figure 4E), and the numbers of positive cells for col2a1a-mcherry were significantly decreased in the ethmoid plate at 72 hpf (Figures 4F,G), indicating a suppression of palatogenesis.

### ZFAS1 Was Distributed in Both the Nucleus and Cytoplasm of Human Oral Keratinocyte Cells and Human Umbilical Cord Mesenchymal Stem Cells

**FIGURE 5:**
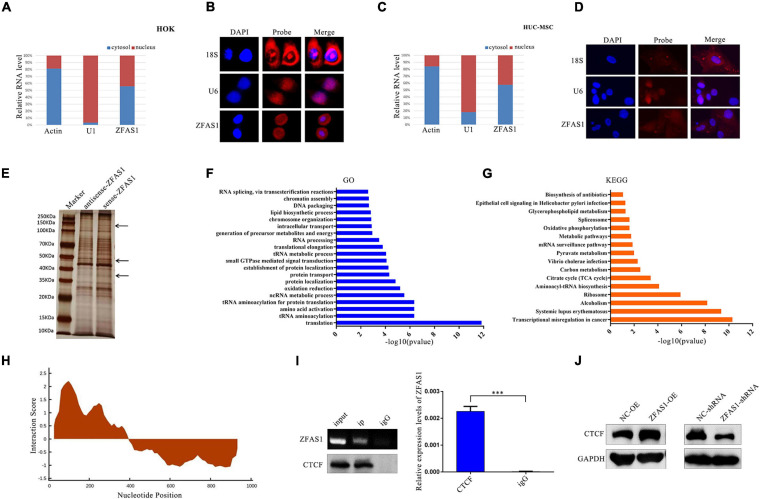
ZFAS1 regulated the protein level of CCCTC-binding factor (CTCF) through binding with CCCTC-binding factor (CTCF) protein. **(A,C)** Nucleus cytoplasm separation indicated that ZFAS1 was distributed in both the nucleus and cytoplasm of human oral keratinocyte (HOK) cells and human umbilical cord mesenchymal stem cells (HUC-MSCs). **(B,D)** RNA fluorescence *in situ* hybridization (FISH) assays indicated that ZFAS1 was distributed in both the nucleus and cytoplasm of HOK cells and HUC-MSCs. Left, DAPI was used to stain the nuclei (blue); middle, red fluorescence was from the biotin fusions; right, the merged image. **(E)** Proteins retrieved from the ZFAS1 pull-down assay were analyzed by sodium dodecyl sulfate–polyacrylamide gel electrophoresis (SDS-PAGE). The arrows indicate the unique proteins in the sense-ZFAS1 group compared with antisense-ZFAS1 group. **(F,G)** The gene ontology (GO) and Kyoto Encyclopedia of Genes and Genomes (KEGG) pathways enriched in these proteins from RNA pull-down assay. **(H)** The predicted binding sites of ZFAS1 with CTCF by catRAPID. The interaction score indicated the possibility of binding. **(I)** RNA immunoprecipitation (RIP) assay of ZFAS1 with anti-CTCF antibody. ZFAS1 and CTCF interaction in CTCF-RNA precipitates was revealed by nucleic acid gel (top left panel) and qPCR assays (right panel). Bottom left panel indicates that the CTCF protein was pulled down by anti-CTCF antibody. **(J)** ZFAS1 positively regulated CTCF protein levels by western blotting (WB). Values are mean ± SEM, *n* = 3, ***P < 0.0001.

The subcellular localization of ZFAS1 determines its action mode; therefore, to further explore the underlying mechanism of ZFAS1 affecting phenotypes above, cell localization testing is necessary. Nuclear–cytoplasmic separation assay indicated that ZFAS1 was distributed in both the nucleus and cytoplasm in HOK and HUC-MSCs (Figures 5A,C), which was verified by RNA-FISH using ZFAS1-probe (Figures 5B,D).

### ZFAS1 Affected the Protein Level of CCCTC-Binding Factor Through Binding to CCCTC-Binding Factor Protein

Growing evidence indicated that lncRNAs often exert their functions by binding to protein partners. To identify potential proteins that interact with ZFAS1, *in vitro* RNA pull-down assay of ZFAS1 was performed in HOK cells, followed by mass spectrometry (Figure 5E). Then GO and KEGG pathway analyses indicated that the proteins in mass spectrometry were enriched in the pathways related to chromosome organization, DNA packaging, transcription, translation, and so on (Figures 5F,G). One of these proteins was CTCF, a transcriptional regulator with diverse functionality in disease through transcriptional activation or repression. ZFAS1 was predicted to have a high probability to directly interact with CTCF at 101–152 nucleotides of ZFAS1 using an online tool catRAPID (Figure 5H). RIP assay was used to verify the binding ability of ZFAS1 to CTCF. In comparison with the IgG group, ZFAS1 enrichment was significantly upregulated in the CTCF group (Figure 5I). Moreover, the protein level of CTCF was significantly upregulated after ZFAS1 overexpression and downregulated after ZFAS1 knockdown (Figure 5J).

### ZFAS1 Inhibited WNT/β-Catenin Signaling Pathway *via* Suppressing WNT4 Expression Levels by Binding With CCCTC-Binding Factor

**FIGURE 6:**
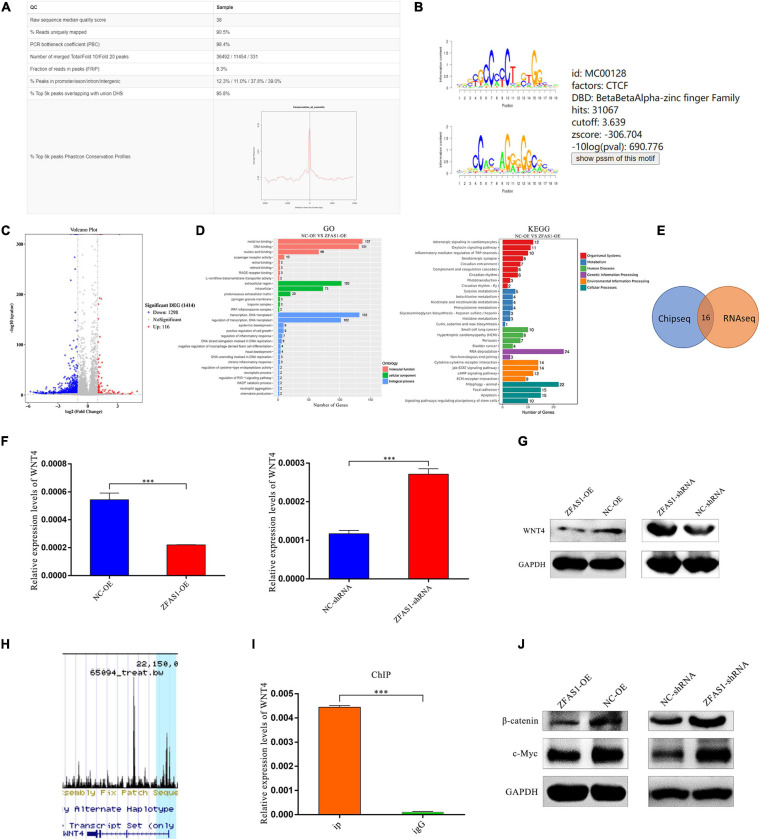
ZFAS1 inhibited WNT/β-catenin signaling pathway *via* suppressing WNT4 expression levels by binding with CCCTC-binding factor (CTCF). **(A)** The quality report of chromatin immunoprecipitation sequencing (ChIP seq) of CTCF mined in the Cistrome Data Browser. **(B)** The annotated binding motif of CTCF from the mined ChIP seq. **(C)** The volcano plot of differentially expressed genes from the RNA seq of human oral keratinocyte (HOK) cells overexpressing ZFAS1. The fold change threshold is 1.5, and *P*-value ≤ 0.05. The red dots indicate upregulated genes, the blue dots indicate downregulated genes, and the gray dots indicate non-differentially expressed genes. **(D)** The gene ontology (GO) and Kyoto Encyclopedia of Genes and Genomes (KEGG) pathways enriched in differentially expressed genes. Length of the bar represents the amount of differentially expressed genes enriched in the pathway. **(E)** The integrated analysis of ChIP seq and RNA seq. The intersection region indicates the merged genes of them. **(F)** ZFAS1 negatively regulated expression levels of *WNT4* by qPCR assay. **(G)** ZFAS1 negatively regulated protein levels of WNT4 by western blotting (WB) assay. **(H)** The peak of WNT4 from the ChIP seq of CTCF. The blue highlight indicates the peak. **(I)** The binding capacity of CTCF to *WNT4* was measured by ChIP qPCR assay. **(J)** ZFAS1 negatively regulated protein levels of β-catenin and c-Myc by WB assay. Values are mean ± SEM, *n* = 3, ***P < 0.0001.

To further investigate the downstream genes of CTCF, RNA seq and ChIP seq analyses were performed in HOK cells. Numerous potential target genes of CTCF were identified from the ChIP seq data (GSM2026782) in Cistrome Data Browser (Figures 6A,B). RNA seq showed that there were 1,414 differentially expressed genes in HOK cells overexpressing ZFAS1 compared with NC, and then GO and KEGG pathway analyses revealed that these genes were enriched in “Regulation of transcription,” “DNA binding” and “Focal adhesion,” “Signaling pathways regulating pluripotency of stem cells,” “RNA degradation,” and so on (Figures 6C,D).

Among the 16 possible target genes by integrated analysis of RNA seq and ChIP seq (Figure 6E), *WNT4*, which belongs to the WNT/β-catenin signaling pathway related with CLP, was selected for further research. The qPCR assay revealed that overexpressing ZFAS1 downregulated mRNA levels of WNT4, and knockdown of ZFAS1 upregulated mRNA levels of WNT4 (Figure 6F), which was validated in protein levels by western blotting (WB) (Figure 6G). Based on the ChIP seq of CTCF collected by the Cistrome Data Browser and its visualization window, there was a peak near the regulatory region of *WNT4* (Figure 6H). ChIP qPCR results revealed that CTCF directly bound to the predicted binding sites of *WNT4* (Figure 6I). Moreover, WB results indicated that upregulating ZFAS1 significantly also decreased β-catenin and c-myc expression levels, which were important molecules in the WNT/β-catenin signaling pathway, consistent with the tendency in cells downregulating ZFAS1 (Figure 6J). In summary, we suggested that ZFAS1 overexpression inhibited the WNT/β-catenin signaling pathway through suppressing WNT4 by interacting with CTCF.

### SP1 Regulated Expression Levels of ZFAS1 *via* Binding to the E1 Region of ZFAS1 Promoter

**FIGURE 7:**
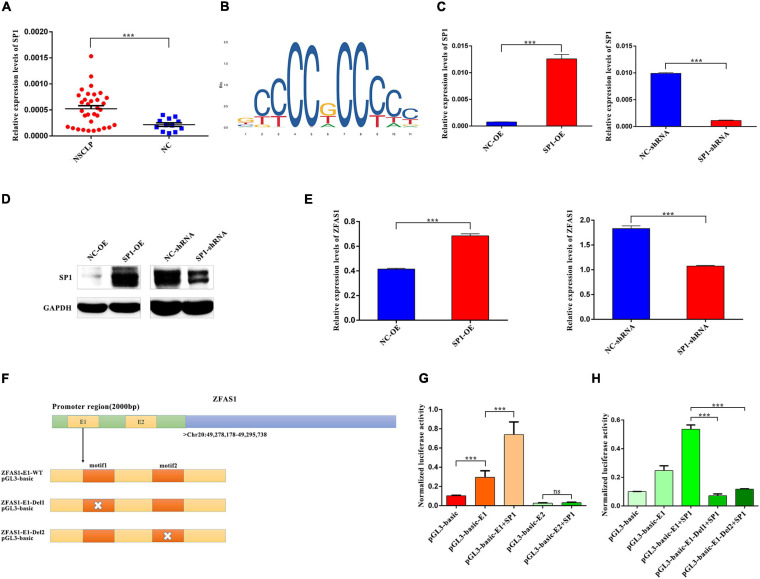
SP1 regulated expression levels of ZFAS1 *via* binding to the E1 region in the promoter of ZFAS1. **(A)** SP1 was upregulated in tissues from non-syndromic cleft lip and palate (NSCLP) compared with NC tissues by qPCR assay. **(B)** The annotated DNA binding motif of ZFAS1 promoter with SP1 in JASPAR database. **(C,D)** The qPCR and western blotting (WB) assays revealed that SP1 was significantly upregulated and downregulated in human oral keratinocyte (HOK) cells. **(E)** SP1 positively regulated expression levels of ZFAS1 by qPCR assay. **(F)** The schema of ZFAS1 for the construction of luciferase reporter plasmids. The orange regions indicate the motif of E1 region in the promoter of ZFAS1; the white cross indicates the deletion of the motif. **(G)** Dual luciferase reporter assay revealed that SP1 significantly increased ZFAS1 promoter activity *via* binding to E1 region instead of E2 region. Compared with the “pGL3-basic-E1” group, the “SP1+pGL3-basic-E1” group showed a higher luciferase activity. **(H)** Dual luciferase reporter assays revealed that deletion of motif1/motif2 in E1 region destroyed the binding of SP1 and E1 region. Compared with the “pGL3-basic-E1 group+SP1 group,” both the “pGL3-basic-E1-Del1+SP1” group and “pGL3-basic-E1-Del2+SP1” showed a significant decrease in the luciferase activity. Values are mean ± SEM, *n* = 3, ***P < 0.0001.

Transcription factors binding to motifs located at genes’ promoter region plays a critical role in their transcription. To ascertain the underlying cause for the elevated expression of ZFAS1 in NSCLP, PROMO database was used to predict the potential transcription factors that possibly bound to the promoter region of ZFAS1. SP1 was selected, and qPCR results revealed that SP1 was upregulated in NSCLP (Figure 7A). And the binding motif in promoters of ZFAS1 was predicted by JASPAR database (Figure 7B). In order to make clear the regulatory relationship between SP1 and ZFAS1, SP1 was successfully upregulated and downregulated in HOK cells, respectively (Figures 7C,D). Moreover, the overexpression of SP1 significantly upregulated the expression of ZFAS1 in HOK cells (Figure 7E). Subsequent bioinformatics analyses in JASPAR database also predicted two binding domains (E1 and E2) of ZFAS1 with SP1 (Figure 7F). To determine the transcriptional activation of SP1 on the ZFAS1, HOK cells were co-transfected with the ZFAS1 promoter luciferase reporter (pGL3-basic-ZFAS1-E1/E2) and SP1. Dual-luciferase assay revealed that SP1 markedly increased ZFAS1 promoter activity *via* binding to the E1 region instead of the E2 region (Figure 7G). Whether depleting the motif1 or motif2 in E1 region, they both destroyed the binding of SP1 and E1 region, thus leading to the suppression of ZFAS1 transcription (Figure 7H).

## Discussion

Long non-coding RNAs have been reported to play a critical role in embryonic development and related disorders (Grote et al., 2013; Alvarez-Dominguez and Lodish, 2017). However, little was known about the function of lncRNA on lip and the palate morphogenesis or CLP. For a better understanding of lncRNA expression profiles in NSCLP, we performed lncRNA sequencing in tissues from NSCLP and NC and then identified many lncRNAs that were aberrantly expressed in NSCLP, which were enriched in “epidermis development,” “skin development,” “protein targeting,” “Ribosome,” “Focal adhesion,” and so on by GO and KEGG analyses. Among these differentially expressed lncRNAs, we focused on ZFAS1, which showed a significant upregulation in NSCLP. ZFAS1 has been reported to be involved in several cancers, including hepatocellular carcinoma (Li et al., 2015), osteosarcoma (Liu et al., 2017), breast cancer (Askarian-Amiri et al., 2011), and colorectal cancer (Zeng et al., 2018), by modulating cell proliferation and migration. ZFAS1 has also been shown to be dysregulated in the cartilage of osteoarthritis (Xiao et al., 2019). Nevertheless, none of the reports declared the role of ZFAS1 on the lip and palate morphogenesis or CLP. Lip and palate morphogenesis is involved in proliferation, migration, apoptosis of a variety of cells, and chondrogenic differentiation of precursor cells, such as MSCs.

To figure out the relationship of upregulated ZFAS1 and NSCLP, two cell models and zebrafish model were selected for further experiments *in vitro* and *in vivo*. One precursor cell was HUC-MSCs, which can differentiate into chondrocytes (Brown et al., 2019). Another mature cell line was HOK cells from oral mucosa, which was used to study the pathogenesis of NSCLP in previous studies (Zhou et al., 2018; Li et al., 2020). The zebrafish palate consists of cells derived from the frontonasal and maxillary domain, which is similar to the palatogenesis of mammals (Duncan et al., 2017). So zebrafish is also a proper study model for mammalian palate and CLP.

*In vitro*, the overexpression of ZFAS1 significantly inhibited cell proliferation and migration in HUC-MSCs and HOK cells and also suppressed chondrogenic differentiation of HUC-MSCs. *In vivo*, upregulating ZFAS1 significantly decreased cell proliferation and numbers of chondrocyte in the zebrafish ethmoid plate. These abnormal cell and zebrafish phenotypes were all associated with NSCLP (Kang and Svoboda, 2005; Jiang et al., 2006; Shull et al., 2020). Therefore, we speculated that upregulation of ZFAS1 may contribute to NSCLP through disturbances in these cell processes, which was consistent with the function of ZFAS1 in previous studies (Askarian-Amiri et al., 2011; Li et al., 2015; Liu et al., 2017). In order to find out the molecular pathways underlying ZFAS1 affecting the phenotypes above, we searched for its molecular chaperones. Increasing studies have reported that lncRNAs could recruit proteins and regulate the downstream pathway (Wang et al., 2019). CTCF was identified as one of the binding proteins of ZFAS1 by RNA pull-down and RIP. What is more, there was a positive regulation of ZFAS1 on CTCF protein level. The change of protein level was mainly due to the changes in the process of its synthesis or degradation. LncRNA could be involved in the protein stability through ubiquitylation (Zhang et al., 2015). CTCF was also reported to be interacted with ubiquitin (Qi et al., 2012). Moreover, among the proteins pulled down by ZFAS1, there was an E3 ubiquitin-protein ligase. So we speculated that ZFAS1 might regulate the protein level of CTCF through ubiquitin proteasome pathway. CTCF is a highly conserved 11 zinc finger DNA-binding domain protein (Franco et al., 2014) and is involved in diverse regulatory functions, including transcriptional activation or repression, modulation of histone modifications, and blocking of enhancer–promoter communication (Huang et al., 2013; Roy et al., 2018). Given the characteristics of CTCF, its target gene *WNT4* was determined by ChIP seq and RNA seq. Further assays revealed that upregulated ZFAS1 inhibited the expression levels of *WNT4* through the binding with CTCF. WNT4 is a member of WNT protein family, and the WNT molecules activate intracellular signals through specific receptors, subsequently modulating various cell processes, including proliferation, migration, differentiation, and apoptosis (Hu et al., 2013a). They exert activations through three WNT signaling pathway, including canonical WNT/β-catenin, WNT/calcium, and planar cell polarity pathways (Song et al., 2009). The WNT/β-catenin pathway is a major signaling pathway that modulates facial morphogenesis especially the lip and palate (Hu et al., 2013b; Jin et al., 2020). A series of phenotype about craniofacial development were observed in β-catenin conditional knockout and Tcf/Lef knockout embryos (Brault et al., 2001; Brugmann et al., 2007). Canonical WNT signaling in oral epithelium was proved to play a dynamic role in tongue and palate development (Lin et al., 2011). In our present study, β-catenin and c-Myc, key components in the canonical WNT/β-catenin pathway, were inhibited when ZFAS1 was overexpressed. So we speculated that the WNT/β-catenin pathway was the underlying mechanism of ZFAS1 regulating cell proliferation, migration, and chondrogenic differentiation in NSCLP.

To further explore the reason of ZFAS1 upregulation in NSCLP, the transcription factors that ZFAS1 may bind to were predicted, and we focused on SP1. It was reported to be a key transcription factor regulating expression levels of lncRNAs (Xu et al., 2015; Wang et al., 2017). Moreover, SP1 could combine with the promoter of ZFAS1 to regulate ZFAS1 in colorectal cancer (Chen et al., 2018). Following luciferase reporter assays, SP1 markedly increased ZFAS1 promoter activity *via* binding to E1 region in the promoter of ZFAS1 instead of E2 region. Moreover, SP1 was upregulated in tissue samples from patients with NSCLP, which was in accordance with ZFAS1. Therefore, we have made a conclusion that SP1 could promote the transcription of ZFAS1 by binding to the E1 region in the promoter of ZFAS1 in NSCLP.

## Conclusion

In conclusion, we have demonstrated that lncRNA ZFAS1 was involved in NSCLP for the first time. The upregulation of ZFAS1 in NSCLP mediated by SP1 inhibited cell proliferation, migration, and chondrogenic differentiation through inactivation of the WNT/β-catenin signaling pathway, which was due to the interaction of ZFAS1 and CTCF to regulate the expression of WNT4. However, further studies are needed to declare the detailed mechanism. Moreover, if the primary cell lines from lip and palate are used for *in vitro* study, the conclusion will be more credible.

## Data Availability Statement

The datasets presented in this study can be found in online repositories. The names of the repository/repositories and accession number(s) can be found below: SRA data database, accession: PRJNA700692; PRJNA700786.

## Ethics Statement

The studies involving human participants were reviewed and approved by the Institutional Research Ethics Committee of the Children’s Hospital of Fudan University, Shanghai, China (2016-121). Written informed consent from the participants’ legal guardian/next of kin was not required to participate in this study in accordance with the national legislation and the institutional requirements. The animal study was reviewed and approved by the Research Ethics Committee of the Children’s Hospital of Fudan University, China, approved and monitored all zebrafish procedures following the guidelines and recommendations outlined by the Guide for the Care and Use of Laboratory Animals (2016-121).

## Author Contributions

JM, DM, and CT were responsible for the idea, project design, and concept of the manuscript. SC and YG performed bioinformatics analysis. SC, ZJ, MC, DW, and TW collected the clinical samples and information. SC, MY, BZ, PW, and YX performed the clinical sample detection and experiments. JM, SC, DM, and CT wrote, edited, and revised the manuscript. All authors read and approved the manuscript.

## Conflict of Interest

The authors declare that the research was conducted in the absence of any commercial or financial relationships that could be construed as a potential conflict of interest.

## Footnotes

1 http://david.abcc.ncifcrf.gov/

2 https://www.ncbi.nlm.nih.gov/

3 http://cistrome.org/db/#/

## Supplementary Material

Supplementary Figure 1 The qPCR results of another 9 lncRNAs in tissues from NSCLP compared with NC tissues. Values are mean ± SEM, *n* = 3, ns: no significance, ^∗^*P* < 0.05, ^∗∗^*P* < 0.01, ^∗∗∗^*P* < 0.0001.

Supplementary Figure 2 HUC-MSCs were identified with a specific panel of cell surface markers of MSC by FACS. **(A)** The FACS results of HUC-MSCs stained with nothing; **(B)** the FACS results of HUC-MSCs identified by negative markers(CD34; CD11b; CD19; CD45; HLA-DR); **(C–E)** the FACS results of HUC-MSCs identified by positive marker(CD90; CD105; CD73); **(F)** The statistical results of **A–E**.

Supplementary Figure 3 ZFAS1 did not affect cell apoptosis in HOK cells. **(A,B)** The cell apoptosis results in HOK cells with upregulating ZFAS1. **(C)** The statistical results of **A,B**. **(D,E)**. The cell apoptosis results in HOK cells with downregulating ZFAS1. **(F)** The statistical results of **D,E**.
