# Supplementary figures and images for: SP1-Mediated Upregulation of Long Noncoding RNA ZFAS1 Involved in Non-syndromic Cleft Lip and Palate via Inactivating WNT/β-Catenin Signaling Pathway
Source: Front Cell Dev Biol. 2021 Jun 29;9:662780. doi: 10.3389/fcell.2021.662780 (PMC8275830; doi:10.3389/fcell.2021.662780)

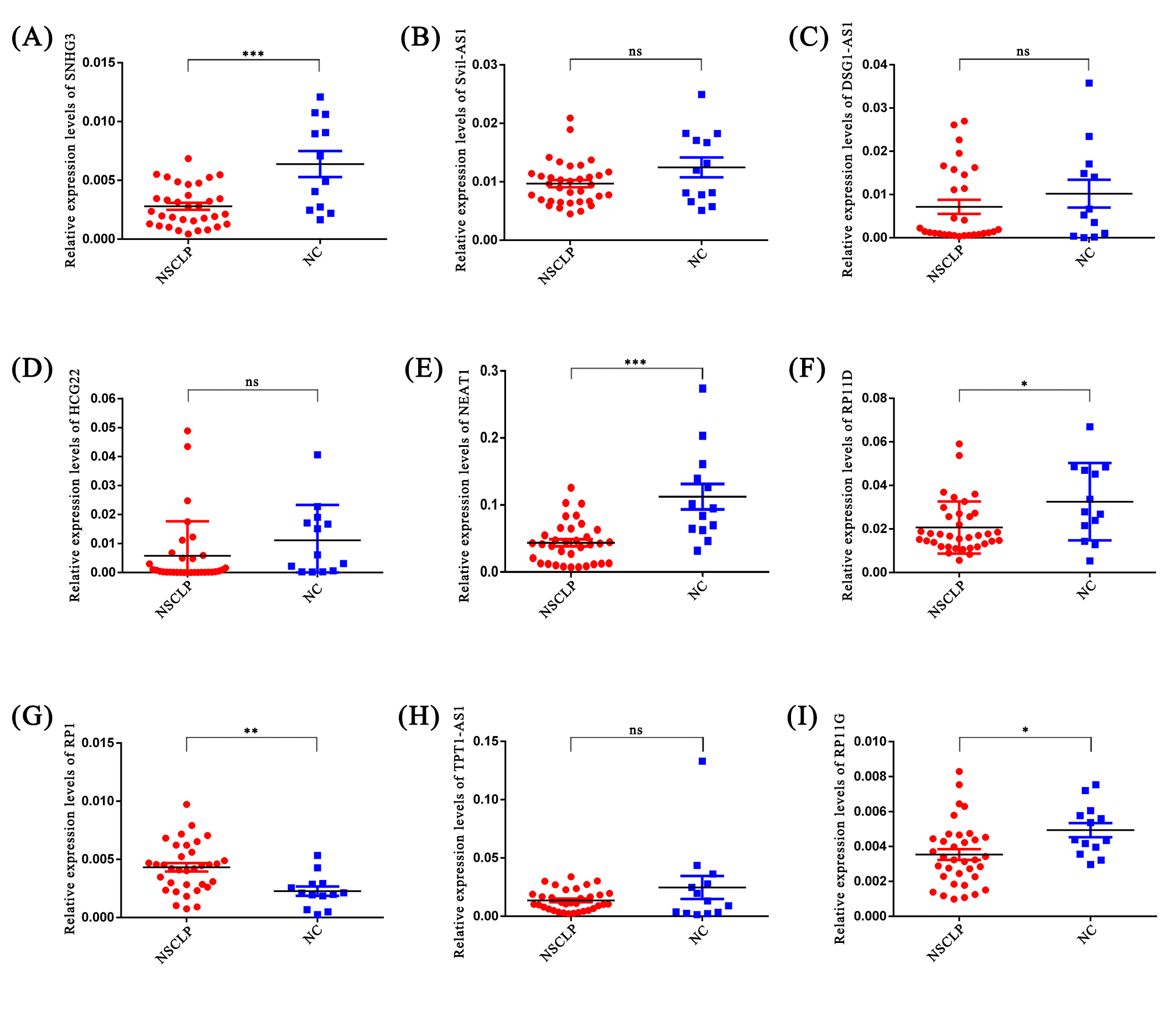

Supplement: Supplementary Figure 1 — The qPCR results of another 9 lncRNAs in tissues from NSCLP compared with NC tissues. Values are mean ± SEM, n = 3, ns: no significance, ∗P < 0.05, ∗∗P < 0.01, ∗∗∗P < 0.0001. [file Image_1.TIF]

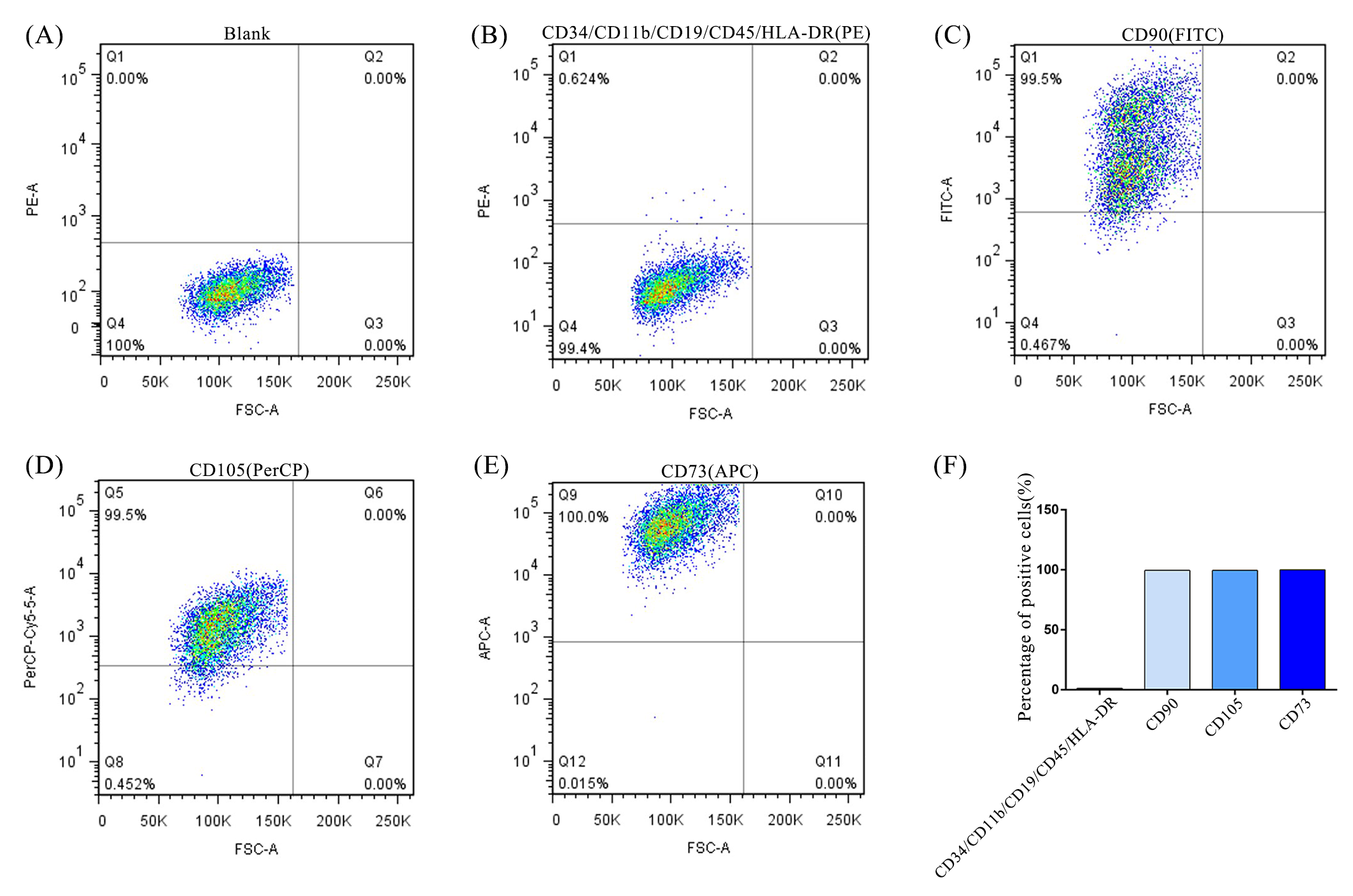

Supplement: Supplementary Figure 2 — HUC-MSCs were identified with a specific panel of cell surface markers of MSC by FACS. (A) The FACS results of HUC-MSCs stained with nothing; (B) the FACS results of HUC-MSCs identified by negative markers(CD34; CD11b; CD19; CD45; HLA-DR); (C–E) the FACS results of HUC-MSCs identified by positive marker(CD90; CD105; CD73); (F) The statistical results of A–E. [file Image_2.TIF]

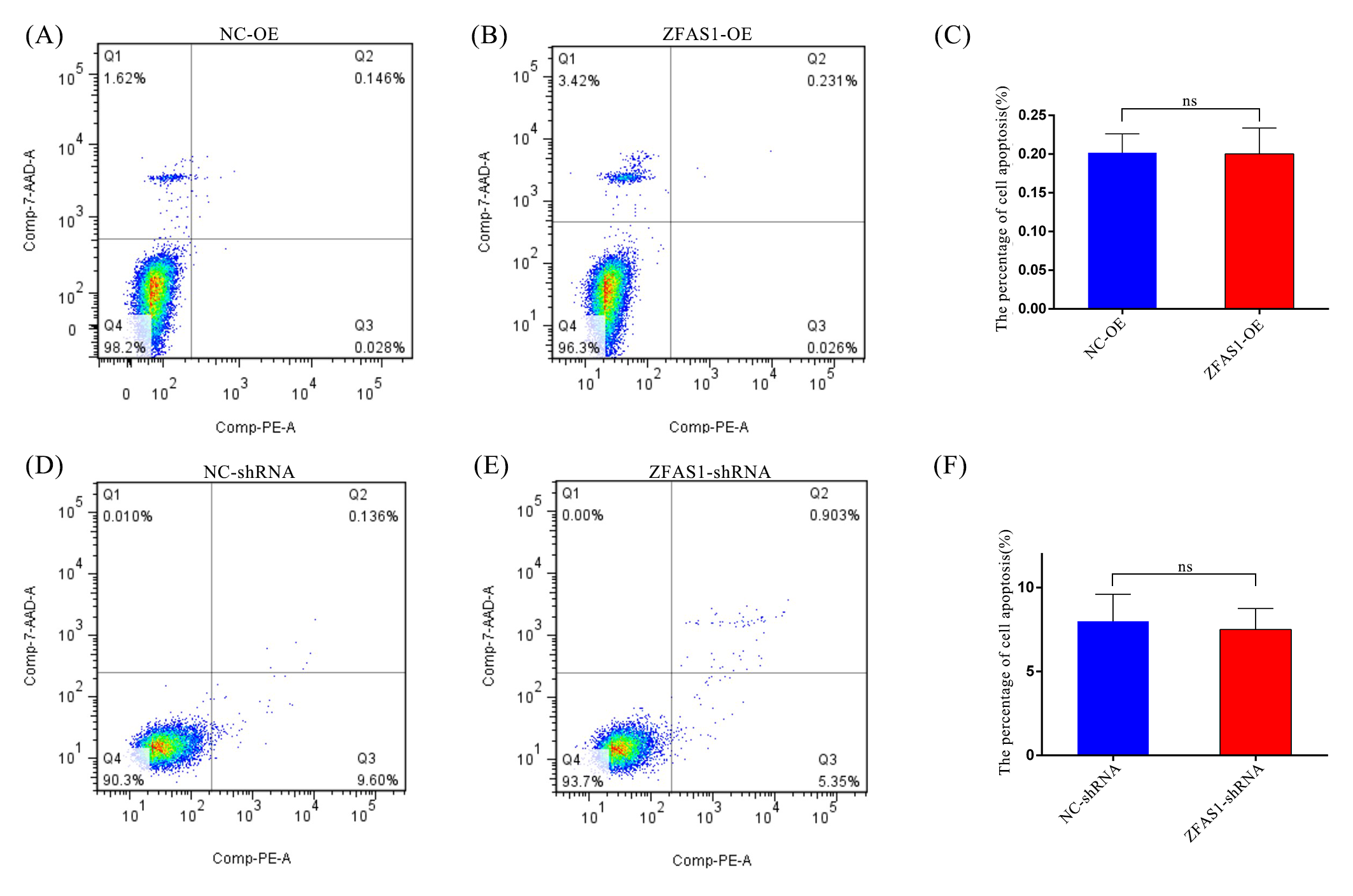

Supplement: Supplementary Figure 3 — ZFAS1 did not affect cell apoptosis in HOK cells. (A,B) The cell apoptosis results in HOK cells with upregulating ZFAS1. (C) The statistical results of A,B. (D,E). The cell apoptosis results in HOK cells with downregulating ZFAS1. (F) The statistical results of D,E. [file Image_3.TIF]
